# 3D Printed In Vitro Dentin Model to Investigate Occlusive Agents against Tooth Sensitivity

**DOI:** 10.3390/ma14237255

**Published:** 2021-11-27

**Authors:** Shiva Naseri, Megan E. Cooke, Derek H. Rosenzweig, Maryam Tabrizian

**Affiliations:** 1 Department of Biomedical Engineering, McGill University, Montreal, QC H3A 2B4, Canada; shiva.naseri@mail.mcgill.ca; 2 Department of Surgery, McGill University, Montreal, QC H3G 1A4, Canada; megan.cooke@mail.mcgill.ca (M.E.C.); derek.rosenzweig@mcgill.ca (D.H.R.)

**Keywords:** 3D printing, dentin, occlusive particles, tooth sensitivity

## Abstract

Tooth sensitivity is a painful and very common problem. Often stimulated by consuming hot, cold, sweet, or acidic foods, it is associated with exposed dentin microtubules that are open to dental pulp. One common treatment for tooth hypersensitivity is the application of occlusive particles to block dentin microtubules. The primary methodology currently used to test the penetration and occlusion of particles into dentin pores relies upon dentin discs cut from extracted bovine/human teeth. However, this method is limited due to low accessibility to the raw material. Thus, there is a need for an in vitro dentin model to characterize the effectiveness of occlusive agents. Three-dimensional printing technologies have emerged that make the printing of dentin-like structures possible. This study sought to develop and print a biomaterial ink that mimicked the natural composition and structure of dentin tubules. A formulation of type I collagen (Col), nanocrystalline hydroxyapatite (HAp), and alginate (Alg) was found to be suitable for the 3D printing of scaffolds. The performance of the 3D printed dentin model was compared to the natural dentin disk by image analysis via scanning electron microscopy (SEM), both pre- and post-treatment with occlusive microparticles, to evaluate the degree of dentinal tubule occlusion. The cytocompatibility of printed scaffolds was also confirmed in vitro. This is a promising biomaterial system for the 3D printing of dentin mimics.

## 1. Introduction

Dentin contains thousands of open-ended microtubules to ensure its permeability. This permeability is essential to support the physiology and reaction patterns of the pulp–dentin organ. Nutrients and impulses are transported from the pulp via the odontoblast process, while the contents of its tubules maintain the dentin as a vital tissue. When dentin microtubules are exposed to the oral environment [1] through receding gums, loss of the cementum, and smear layers and tooth wear [2], it results in hypersensitivity [3,4].

Dentin hypersensitivity is a very common dental problem characterized by short and sharp pain. It arises in response to stimuli, typically thermal (hot or cold), evaporative, tactile, osmotic (sweet or salty), chemical (acidic or basic), or electrical, with no effective remedy [5,6]. Common short-term treatments include applying fluoride gels, rinses, or varnishes that can be applied to sensitive areas of the teeth at regular intervals to help strengthen the tooth, or tooth surface treatment with occluding agents [7,8]. A current area of interest to reduce dentin sensitivity is decreasing tubule permeability through occlusion [9]. A number of studies have shown that dentin sensitivity is reduced when the dentinal tubules are occluded [10,11,12].

The current gold standard to determine the penetration and adhesion of occlusive particles into dentin pores is to use dentin discs cut from extracted bovine/human teeth and to visualize the treated dentin disks under scanning electron microscopy (SEM) [13]. An important limitation of this method is the low supply of the raw material [14]. Thus, there is a need to develop an in vitro method/model of dentin that allows for the investigation of different occlusion agents. Additionally, developing a 3D printed cytocompatible dentin-mimic, with the ability to maintain the bone cell phenotype, is highly beneficial to concurrently assess the effects of occlusive particles on cell behaviour.

Both adhesion onto the dentin surface and penetration into the tubules are important properties for occlusion agents. When considering the former, a material mimic should be of similar composition to the native dentin. Dentin is a biological composite containing 65–75 wt% of hydroxyapatite (HAp), 15–20 wt% of type I collagen (Col), and 10–15 wt% of dentinal fluid, mainly water [15,16]. To evaluate the penetration of occlusive agents into tubules, the model system must have a comparable morphology and microstructure [15,16]. Importantly, matching the native porosity and a pore size of 3–5 µm are essential to achieve a dentin-like structure [17].

Three-dimensional printing of polymer filaments using techniques such as fused deposition modelling are well established to produce structures with defined pore sizes and geometry. The printing of gels and pastes, however, is more challenging due to their relaxation prior to solidification [18]. Printability of a dentin-like paste depends on the rheological profile of the ink; it should be a yield stress fluid that is shear thinning, with minimal thixotropy [19]. Print parameters such as extrusion pressure, print speed, and needle size and geometry can also be used to tailor print characteristics [20]. Several attempts have been made to develop a suitable bioink for dentin tissue regeneration using 3D printing.

A few recent approaches have attempted to mimic the dentin structure [21]. Recently, Han et al. reported a fibrin-based bioink composed of fibrinogen, gelatin, hyaluronic acid, and glycerol for 3D printing of human dental pulp stem cells to construct a 3D dentin–pulp complex [22]. It was shown that the printed scaffold had a pore diameter of 2–4 μm similar to that of a human dentinal tubule [22]. In another study, a novel bioink consisting of a hydrogel blend of the soluble and insoluble fractions of the dentin extracellular matrix and alginate was developed to fabricate 3D printed scaffolds for regenerative dentistry [23]. It should be noted that the smallest pore size of the printed structure was around 2 mm, much larger than the size of natural dentin tubes. Wu et al. studied the potential of 3D printed scaffolds composed of a polycaprolactone/mineral trioxide aggregate to support the regeneration of dentin and periodontal tissue [24]. This scaffold had a pore size of 200 μm, about 60–100 times larger than native dentin. In addition to the pore size, the composition lacked the presence of collagen as a main component for biological interactions. Overall, the above-mentioned studies do not mimic or satisfy the natural dentin composition and microstructure. To the best of our knowledge, no 3D printed dentin model has been proposed in the literature. Thus, the development of a novel 3D printed construct similar to natural dentin has a great benefit for researchers to effectively develop and evaluate the potential of occlusive agents. Additionally, the 3D printing of hard tissues such as bone and dental tissues is a favourable fabrication technique and a robust method to develop a customized tissue suitable for specific patient needs [25].

In this study, a compositionally and microstructurally appropriate dentin mimic was produced using a collagen–hydroxyapatite–alginate ink. The addition of alginate enabled rapid ionic crosslinking following printing to ensure shape fidelity. Extrusion-based 3D printing was used with specific layer-shifting to produce pores with tubule-like diameters. Following lyophilisation, the occlusion of tubules was investigated as well as the cytocompatibility of the dentin mimic.

## 2. Materials and Methods

Calcium nitrate tetrahydrate (Fisher, Hampton, NH, USA), ammonium phosphate (Fisher, USA), ammonium hydroxide (Sigma Aldrich, Oakville, ON, USA), hydroxyapatite (Sigma Aldrich, Oakville, ON, Canada), atelo-collagen solution (Advanced BioMatrix, Carlsbad, CA, USA), sodium hydroxide (Fisher, USA), and sodium alginate (DuPont, Wilmington, DE, USA) were utilized for ink preparation. Calcium chloride (Fisher, Schwerte, Germany) and glutaraldehyde (Sigma-Aldrich, Schnelldorf, Germany) were used as crosslinking agents. Thymol (Fisher, USA), ethanol (Fisher, USA), and citric acid (Sigma Aldrich, USA) for dentin disk preparation and polystyrene microspheres (Sigma Aldrich, USA) for the occlusion test were utilized. For cell study, bone marrow-derived stem cells (Lonza, Bend, OR, USA), high glucose DMEM (Gibco, Thermo Fisher, Ottawa, ON, Canada), fetal bovine serum (Gibco, Canada), 1% penicillin–streptomycin (Gibco, Canada), ascorbic acid (Sigma Aldrich, Oakville, ON), dexamethasone (Thermo Fisher, Waltham, MA, USA), and β-glycerophosphate (Sigma Aldrich, Oakville, ON, Canada) were used.

### 2.1. Nanocrystalline Hydroxyapatite Paste Preparation

Hydroxyapatite was prepared by a solution–precipitation method [26] with calcium nitrate tetrahydrate (Ca(NO_3_)2·4H_2_O) and ammonium phosphate ((NH_4_)2HPO_4_) as precursor materials while ammonium hydroxide (NH_4_OH) was used for pH adjustment. A total of 350 mL of 0.24 M calcium nitrate tetrahydrate was prepared in DI water and adjusted to pH 10. Under constant stirring, 250 mL of 0.29 M ammonium phosphate was added dropwise to the calcium nitrate tetrahydrate while maintaining the pH between 9 and 11. The solution was stirred for 16 h before the precipitate was allowed to sediment. The aqueous phase was removed, and the precipitated HAp paste was collected by centrifugation at 3000 rpm. To remove residual ammonium, the paste was vigorously washed with DI water and centrifuged 5 times.

#### 2.1.1. X-ray Diffraction (XRD)

The preparation of nanocrystalline HAp was confirmed by XRD. Samples were analyzed with a Bruker D8 Advance X-ray diffractometer (Bruker AXSS Inc., Fitchburg, WI, USA) equipped with a CuKα (λ = 0.15406 nm) target set to a power level of 40 mV and 40 mA. Data were collected from 10 to 90 2 theta (°) with 0.02 increments. Phase identification was carried out using X’Pert HighScore Plus (PANalytical, Almelo, The Netherlands). Commercially available synthetic hydroxyapatite nanopowder (Sigma Aldrich Cat no. 677418) with an average particle size of 73 nm was used as a reference for HAp peaks and 3 independent syntheses were investigated for crystallite size, calculated using the Scherrer equation.

#### 2.1.2. Attenuated Total Reflection-Fourier Transform Infrared (ATR-FTIR) Spectroscopy

FTIR spectra of the hydroxyapatite powder were measured using a Spectrum Two FTIR Spectrometer (PerkinElmer, Waltham, MA, USA) in ATR mode. A spectral resolution of 4 cm^−1^ with 16 scans was used for collection of reference spectra over a range of 4000–400 cm^−1^.

#### 2.1.3. Transition Electron Microscopy-Energy Dispersive X-ray (TEM-EDX)

TEM images and EDS analysis were obtained using a Thermo Scientific Talos F200X G2 S/TEM operating at 200 kV. Samples were prepared by diluting and dispersing a very small amount of HAp in mQ water. Carbon-coated copper TEM grids were briefly submerged in the sample and dried before imaging. A 1 min acquisition time was used for the EDS analysis.

#### 2.1.4. Nanoparticle Tracking Analysis (NTA)

A NanoSight NS300 (Malvern Panalytical Ltd., Malvern, UK) was employed to measure the size of HAp nanoparticles. The HAp paste was diluted in DI water to achieve approximately 20 particles per frame. A video of 30 s duration with three repeats was taken, and particle size was analyzed by NTA software (NanoSight Ltd., Salisbury, UK).

### 2.2. Ink Preparation

Bovine Type I atelo-collagen solution at 3 mg/mL was neutralized with 1 M of NaOH and mixed with 2 *w*/*w*% alginate solution and 12 *w*/*w*% HAp paste. The mixture was placed in a dry incubator at 37 °C until sufficient water was evaporated to constitute an 80% weight reduction to render a printable composite paste of 8.6 *w*/*w*% HAp, 1.1 *w*/*w*% collagen, and 0.5 *w*/*w*% alginate.

### 2.3. Ink Rheology

All rheological measurements were performed using an MCR 302 rheometer (Anton Paar, Graz, Austria) with 25 mm stainless steel parallel plates and a gap height of 0.5 mm. A total of 0.5 mL of material was added to the gap and trimmed where necessary before light mineral oil (Sigma-Aldrich, USA) was applied to prevent evaporation during measurements. Shear strain ramps from 0.1 to 100% and 3-step thixotropy measurements at a shear rate of 0.1, 500, and 0 s^−1^ were performed at room temperature.

### 2.4. Scaffold Fabrication

Scaffolds were printed at room temperature using a pneumatically controlled bioprinter (BioX, Cellink, Boston, MA, USA). Layer shifting with a 200 μm offset was used in alternating layers to reduce pore size. The inner diameter of the nozzle was 518 μm and the pneumatic pressure was 40−50 kPa with a print speed of 10–12 mm/s. Immediately after printing, scaffolds were crosslinked with 0.1 M of CaCl_2_ for 1 h and then washed three times with DI water. Scaffolds were then crosslinked with glutaraldehyde for 1 h and again washed three times with DI water to remove remaining crosslinking agent. Finally, scaffolds were freeze dried (samples were frozen at −80 °C for 24 h and freeze dried at −40 °C and 300 mTorr for 24 h), resulting in a composite material of 80 *w*/*w*% HA–10 *w*/*w*% collagen–5 *w*/*w*% alginate that was used for further characterization.

### 2.5. Micro Computed Tomography

Freeze-dried samples were scanned using a SkyScan 1172 (Bruker, Billerica, MA, USA) with a source voltage of 34 kV, current of 210 µA, rotation step of 0.28°, and pixel size of 1.23 µm. Reconstruction was performed using NRecon v1.0 (2018, Bruker) with ring artefact reduction (4), smoothing (2), and beam hardening correction (65%). Quantitative analysis was performed in CTAn v1.18 (2018, Bruker) following thresholding between 110 and 255 to include mineral only and the removal of white speckles smaller than 20 voxels to remove scanning artefacts. While some smaller HAp particles will be removed for the analysis, the chosen scanning parameters were required to ensure the visualization of both the inorganic and organic phases of the material. Organic content was calculated as the difference between the thresholded, mineral-only volume and the total volume of the scaffold.

### 2.6. Natural Dentin Disk Preparation

Extracted teeth were stored in distilled water containing 0.1% thymol and 10% ethanol. To prepare dentin disks, the occlusal part of the enamel was removed with a slow speed diamond saw to expose the dentin, before 1 mm thick dentin disks were cut from the coronal section. To mimic the open dentinal tubules of hypersensitive dentin and to remove the smear layer, the dentin disks were polished by hand using 600 and 1000 grit carbide polishing papers, with circular motions for 10 s each, before they were ultrasonicated in DI water for 2 min. Afterwards they were acid etched using 6% citric acid for 2 min followed by ultrasonication in DI water for 2 min. The prepared dentin discs were stored in 0.1% thymol and 10% ethanol solution before characterization.

### 2.7. Occlusion Test

The fabricated scaffolds were treated with polystyrene microspheres with a diameter of 2–3 µm as simulated occlusive particles. A 10 *w*/*w*% particle suspension was prepared in DI water before 50 µL of suspension was deposited on the top of scaffold for 5 min. Scaffolds were then washed with DI water and dried at room temperature for 24 h before further characterisation.

### 2.8. Sputter Coating and Scanning Electron Microscopy (SEM)

Samples were sputter-coated with a 4 nm layer of Pt (EM ACE600, Leica Microsystems, Buffalo Grove, IL, USA). A FEI Quanta 450 ESEM (FEI Corporation, Hillsboro, OR, USA) was used with an acceleration voltage of 5 kV to image the topography of the native dentin and dentin mimics with and without occlusive particles.

### 2.9. Cytocompatibility and Gene Expression

To confirm the cytocompatibility of the dentin mimics, bone marrow-derived stem cells were seeded at a density of 250,000 cells per scaffold by placing the scaffold in a syringe containing the cell suspension for 2 h, turning every 30 min to ensure homogeneous cell seeding. After 14 days of culture in control media (high glucose DMEM, 10% fetal bovine serum, 1% penicillin–streptomycin) or osteogenic media (as control with ascorbic acid, dexamethasone, and β-glycerophosphate), calcein AM/ethidium homodimer-1 staining (Life Technologies, Carlsbad, CA, USA) was performed to determine the cell morphology and viability on the scaffold. At both 4 and 14 days, RNA was extracted using TriZol reagent (Life Technologies, Carlsbad, CA, USA) for qRT-PCR analysis. Scaffolds were placed in 500 µL of TriZol reagent for 5 min to lyse cells before being centrifuged. The supernatant was aspirated, transferred to a new tube, and used to isolate RNA as per the manufacturer’s instructions while the scaffold was discarded. Briefly, chloroform was used to separate the aqueous RNA phase before it was aspirated, and RNA was precipitated with isopropanol. After centrifugation, the precipitate was washed with 75% ethanol before drying and resuspension in RNAse-free water. RNA concentration was quantified using spectrophotometry (TECAN, Mannedorf, Sweden) before 500 ng was used for cDNA synthesis (qScript kit, QuantaBio, Beverly, MA, USA). RT-PCR was then performed to determine the expression of alkaline phosphatase (ALP), bone sialoprotein (BSP), and runt-related transcription factor 2 (RUNX2) against the housekeeping gene GAPDH (sequences in Table 1, all supplied by Thermo Fisher) with PowerUp SYBR Green Master Mix (Applied Biosystems, Waltham, MA, USA).

### 2.10. Statistical Analysis

Statistical significance of the measured parameters between samples was determined using the Student’s *t*-test at a significance level of *p* < 0.05. Data are presented as mean ± standard deviation.

## 3. Results and Discussion

### 3.1. HAp Characterization

**Figure 1:**
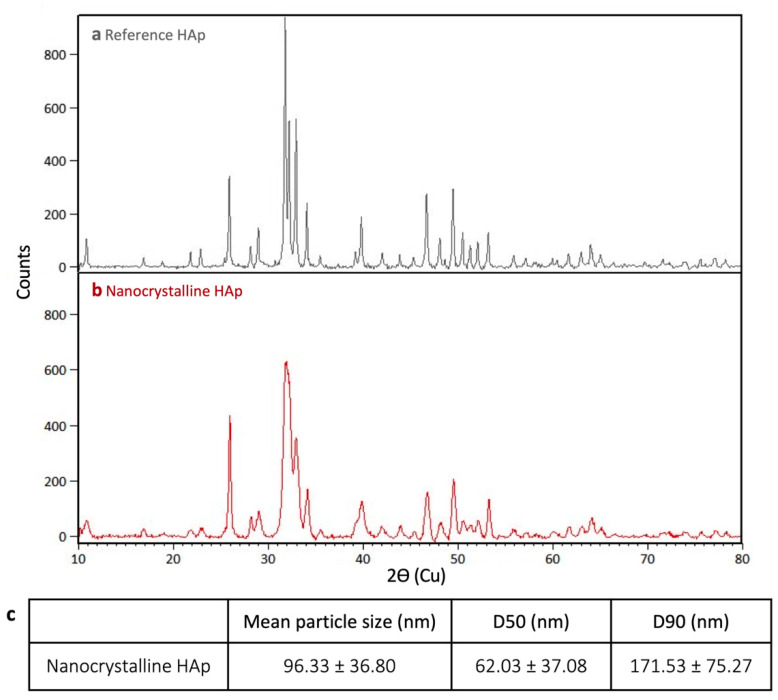
XRD scans of (**a**) reference HAp sample and (**b**) synthesized nanocrystalline HAp. (**c**) Nanoparticle tracking analysis of nanocrystalline HAp particle size (n = 3).

Nanocrystalline HAp was synthesized both to ensure small crystallite sizes and to produce a sol formulation that could be easily incorporated into an ink. Prior to being used in the ink formulation, the synthesized HAp was characterized regarding its crystalline structure and particle size. Compared to the reference sample (Figure 1a), the synthesized nanocrystalline material had broader peaks (Figure 1b, representative scan), particularly between 2ϴ = 31 and 33°, which occluded the peak at 2ϴ = 32.2°, which is normally observed in stochiometric HAp. This poorly-crystalline nature of nanocrystalline HAp has previously been reported by Drouet [27]. Nevertheless, all other characteristic HAp peaks were detected. Crystallite size was measured using the 25.9° peak as it was consistently sharp. The reference material (hydroxyapatite) had a greater crystallite size of 511.6 Å compared to the synthesized nanocrystalline material with a size of 328.5 ± 56.5 Å. The solid phase produced in the precipitation reaction had an average particle size of 96.33 ± 36.8 nm, calculated using nanoparticle tracking analysis. The D50 value (median particle size) was 62.03 ± 37.08 nm and the D90 (90% of particles were smaller than this value) was 171.53 ± 75.27 nm for the synthesized HAp.

**Figure 2:**
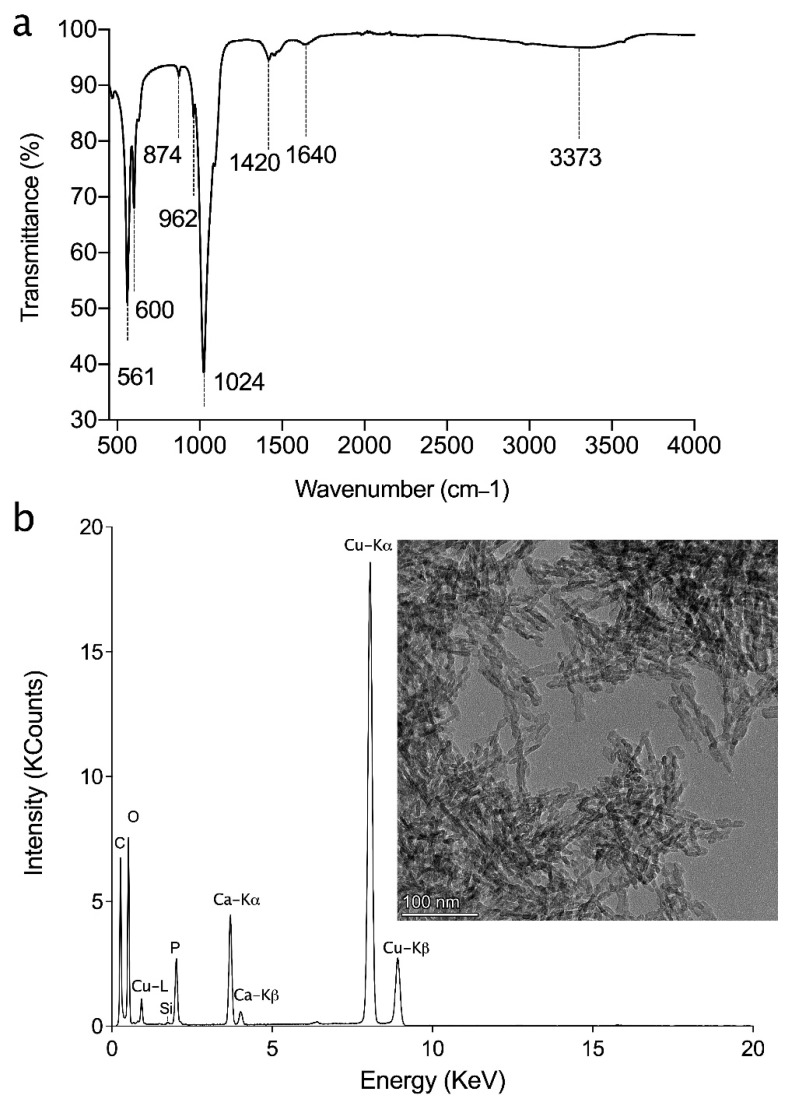
(**a**) FTIR spectra and (**b**) TEM image and its corresponding EDX analysis of synthesized nano HAp powder.

To verify that the residual ammonium was fully removed after the washing step, FTIR was conducted. Figure 2a shows the presence of water between 3700 and 2800 cm^−1^ (peak value at 3373 cm^−1^) and at 1640 cm^−1^ (O–H stretching and H–O–H bending modes of the water molecules, respectively) [28]. Characteristic bands of the PO43− group were detected as well, in particular, v1 stretching mode at 962 cm^−1^, v3 stretching vibration at 1024 cm^−1^, and v4 at 561 cm^−1^ and 600 cm^−1^. Peaks around 874 and 1420 cm^−1^ can be attributed to the presence of carbonate CO_3_^2–^ groups, vibrational mode v2 and v3, respectfully [29,30,31]. No residual nitrate (NO_3_^−^ typical sharp peak at 1384 cm^−1^) and ammonium ions (NH_4_^+^ peak at 1400^−1^) were detected [32,33].

TEM-EDX analysis was conducted (Figure 2b) to verify the morphology and crystal size of HAp particles, which concorded with the XRD and particle size measurements. Further, the EDX analysis indicated that hydroxyapatite crystals were not transformed to the other calcium phosphate phases. There was no evidence of the presence of an amorphous phase [34,35].

### 3.2. Printability of Hap–Collagen Ink

**Figure 3:**
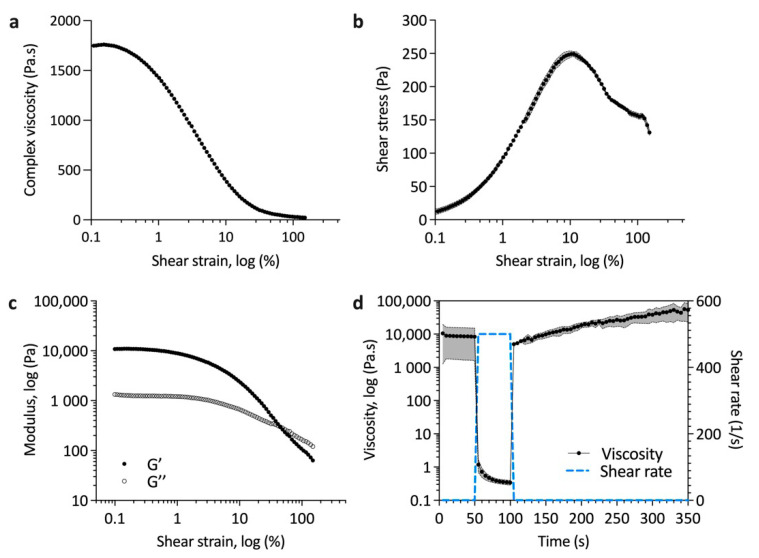
Rheological characterisation of Hap–collagen–alginate ink showing (**a**) yield behaviour with increasing shear strain where complex viscosity was calculated from the storage and loss data, (**b**) yield point and initiation of flow with increasing shear strain, (**c**) storage and loss moduli with respect to increasing shear strain and (**d**) thixotropic recovery after high-rate shearing. Data are plotted as mean ± SD (shaded region), n = 3.

Ideal inks for printing have a yield stress to ensure controlled extrusion, shear thinning flow profiles to allow extrusion through a small orifice, and low thixotropy, such that they quickly recover their pre-shearing elastic modulus or viscosity to ensure a good shape fidelity post-printing [19]. As shown in Figure 3a, the viscosity of the ink decreased with increasing shear strain. The shear thinning properties of the ink are crucial for extrusion-based printing [19,36]. Figure 3b demonstrates clear yielding behaviour at around 10% shear strain, which is important for controlled extrusion. Using oscillatory rheology, the viscous moduli were elucidated with the storage modulus dominating until the flow point at 40% shear strain (Figure 3c). The time an ink needs to return to its initial viscous state after applying high shear stress is important for high fidelity 3D printing [36,37]. Figure 3d shows that that upon removal of a high shear stress (500 s^−1^), the initial viscosity of the ink was recovered in around 10 s, indicating a very low thixotropy. With time there was an increase in viscosity suggesting the aggregation of particles in the ink, which would lend to a further increase of the shape fidelity and stability of the printed part prior to crosslinking.

#### 3D Printed Scaffolds

**Figure 4:**
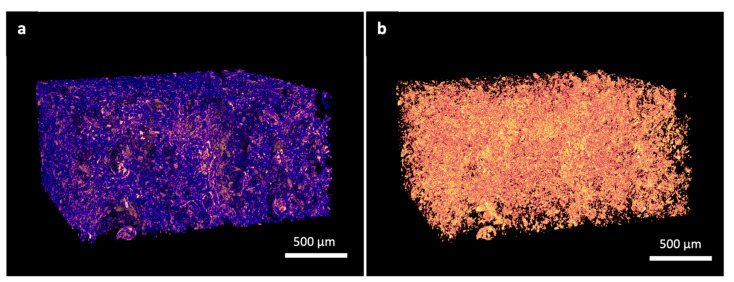
MicroCT reconstruction of representative sample: (**a**) mineral (yellow) and polymeric (blue) components separated by attenuation and overlaid; (**b**) mineral phase shown separately, average mineral content 8.49% (SD 3.65, n = 3).

The printing speed, pressure, and lattice offsets were optimized to achieve the desired pore sizes. Some extrudate swell and relaxation of the ink following printing reduced the shape fidelity, but with increased print speed or decreased pressure, the line width and pore size decreased. In this study, pore sizes mimicking the human dentinal tubule microstructure were achieved with a print speed and pressure of 10–12 mm/s and 40−50 kPa, respectively. With increased printed layers, some full thickness pores were lost. The sublimation of water during freeze drying resulted in both large (250–300 µm) and small (2–5 µm) pores (Figure 4a).

MicroCT scanning, shown in Figure 4b, indicated a homogeneous distribution of mineral throughout the region of interest. The volume of mineral relative to the total volume was calculated as 8.49 ± 3.65%, which is very close to the 8.6 *w*/*w*% stated previously. The decrease is likely due to limitations in detecting all HAp as the minimum observable feature was limited by pixel size (1.23 µm). The total porosity was 78.388% (±1.556%, n = 3), and of this, 99.996% was detected as open porosity, indicating a very high interconnectivity.

**Figure 5:**
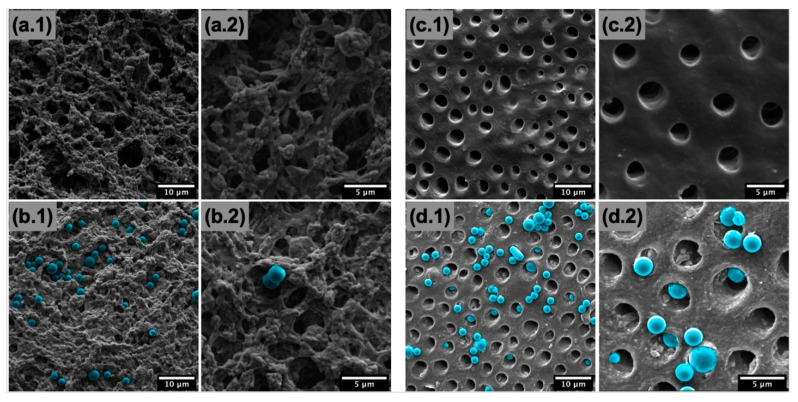
SEM images of (**a.1**,**a.2**,**b.1**,**b.2**) scaffold and (**c.1**,**c.2**,**d.1**,**d.2**) native dentin surfaces before (**a.1**,**a.2**,**c.1**,**c.2**) and after (**b.1**,**b.2**,**d.1**,**d.2**) addition of polystyrene microspheres (blue false colourized) mimicking occlusive particles.

Following crosslinking and freeze drying, scaffolds had good homogeneity and integrity. Structural properties of the 3D printed scaffolds were compared with the topographical microstructure of the natural dentin. The untreated, acid-etched control specimens of dentin exhibited the typical appearance of a microstructure of open tubules (Figure 5(c.1,c.2)). Figure 5(a.1,a.2) shows SEM images of the crosslinked and freeze-dried scaffold. The surface was much rougher than that of the native dentin, but there were clearly pores of similar length scales to the dentin. As shown, the 3D printed dentin-like scaffold had a pore size of 2–4 μm with a homogenous pore distribution (Figure 5(a.1,a.2)), similar to that of a human dentinal tubule (Figure 5(c.1,c.2)).

These results confirmed that the collagen/HA/Alg ink, along with a well-designed printing–freeze drying protocol, could provide a biomaterial for 3D printing of an in vitro dentin model that can mimic a similar microstructure and composition to that of natural dentin tissue.

Polystyrene microspheres were applied as occlusive agents on the surface of acid-etched dentin as the control as well as on the surface of 3D printed scaffolds. Microspheres were visible on the surface of the scaffold and, similarly to the native dentin, some covered the pores and some entered the pores. The pore size and number of open pores for both the 3D printed scaffolds and human dentinal tubules decreased after treatment with polystyrene microspheres as an occlusive agent.

### 3.3. Cytocompatibility of Dentin Mimics

**Figure 6:**
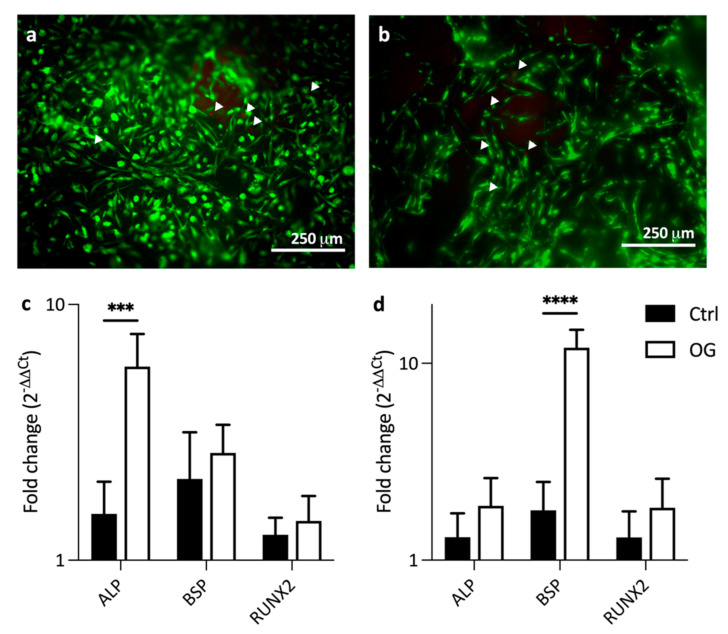
Live/dead staining of bmMSCs cultured on scaffolds for 14 days in (**a**) control and (**b**) osteogenic media demonstrating good cell adhesion and viability. Arrowheads indicate dead cells (stained red). The qRT-PCR analysis of bmMSCs cultured on scaffolds for (**c**) 4 and (**d**) 14 days in either control (Ctrl, black) or osteogenic (OG, white) media shows increased gene expression of ALP, BSP, and RUNX2 in osteogenic media at both time points. Data presented as mean ± SD, n = 3 *** = *p* < 0.0005, **** = *p* < 0.0001.

To confirm that this model can be used to address both the occlusivity and cytocompatibility of therapeutic agents, it is important that the 3D printed scaffold itself is cytocompatible. This was tested using human bone marrow-derived mesenchymal stem cells seeded onto the 3D printed scaffold. After 14 days of culture, cells adhered to the scaffolds and live-dead staining indicated a very good viability, with very few dead cells present (Figure 6a,b). When cultured in osteogenic media, cells expressed increased ALP, BSP, and RUNX2 compared to cells cultured in control media at both 4 and 14 days (Figure 6c,d). ALP expression was significantly higher in the osteogenic condition than controls at day 4 (Figure 6c), while BSP expression was significantly higher after 14 days (Figure 6d). Higher ALP expression at day 4 and BSP expression at day 14 suggest that there is more inhibition of mineralization in the early stage, but also a stronger promotion of osteogenic differentiation in the later stage [38].

## 4. Conclusions

In this study, we aimed to produce a biocompatible ink for the direct printing of a dentin mimic. A collagen–hydroxyapatite–alginate formulation was developed and, with the evaporation of excess water, an extrudable paste was produced. Rheological evaluation showed that the ink fulfilled the required parameters to produce printed constructs of high fidelity. The printing of pores of sizes equivalent to those in native dentin was challenging, but with the sublimation of water, a porous structure was produced. When occlusive particles were added to the surface, they could enter the pores, as in the native dentin surface. Finally, the cytocompatibility of these dentin mimics was confirmed through live/dead staining and the maintenance of the bone cell phenotype when cultured on these scaffolds.

## Figures and Tables

**Table 1:** Primer sequences.

Gene	Forward	Reverse
Alkaline phosphatase (ALP)	AGAACCCCAAAGGCTTCTTC	CTTGGCTTTTCCTTCATGGT
Bone sialoprotein (BSP)	AAGCTCCAGCCTGGGATGA	TATTGCACCTTCCTGAGTTGAACT
Runt-related transcription factor 2 (RUNX2)	TCAGCCCAGAACTGAGAAACTC	TTATCACAGATGGTCCCTAATGGT
Glyceraldehyde 3-phosphate dehydrogenase (GAPDH)	TCCCTGAGCTGAACGGGAAG	GGAGGAGTGGGTGTCGCTGT

## Data Availability

Data available on request. The data presented in this study are available on request from the corresponding author.
